# Improved skill for tracheal intubation using optical stylets through remote training model: a before and after interventional study

**DOI:** 10.1186/s12909-022-03715-x

**Published:** 2022-09-10

**Authors:** Danyun Fu, Weixing Li, Wenxian Li, Yuan Han

**Affiliations:** Department of Anesthesiology, Eye & ENT Hospital of Fudan University, No.83 Fenyang Road, Xuhui District, Shanghai, 200031 China

**Keywords:** Tracheal intubation, Optical stylets, Remote training, Airway management training, COVID-19 pandemic

## Abstract

**Background:**

Conducting on-site, hands-on training during the Coronavirus disease 2019 (COVID-19) pandemic has been challenging. We conducted a before and after interventional study to estimate the efficacy of a new remote hands-on training model for improving the trainees’ tracheal intubation competency using optical stylets.

**Methods:**

Residents or physicians in anesthesiology apartment who have not received the nominated training in tracheal intubation using optical stylets were enrolled. The 4-week training course contains theoretical knowledge along with preclinical and clinical training of optical stylets techniques. Competency of intubation using optical stylets on patients with normal airways was evaluated according to an assessment tool with a maximum score of 29 points based on video recording pre-post training performance. Pre-post questionnaires measured theoretical knowledge and self-efficacy.

**Results:**

Twenty-two participants were included (8 females, 14 men, mean age of 33.5 years). The total score of intubation competency was significantly improved after training from 14.6±3.7 to 25.3±2.6 (*P *< 0.0001). The scores of three subitems (anatomical identification, hand-eye coordination, and optimized intubation condition) were all significantly increased after training (*P *< 0.0001). The total percentage of correct answers in the multiple-choice questionnaire increased from 58.2%±8.2% before training to 85.2%±7.2% shortly after training (*P *< 0.0001). In addition, the self-efficacy score was significantly increased from 2.5±1.2 to 4.4±0.6 (*P *< 0.0001).

**Conclusions:**

The new remote and progressively advanced hands-on training model improved the competency of intubation using optical stylets under the COVID-19 pandemic.

**Supplementary Information:**

The online version contains supplementary material available at 10.1186/s12909-022-03715-x.

## Background

Airway management training is an integral part of an anaesthesiologist’s professional career [1], as delayed or unsuccessful tracheal intubation could cause trauma to the brain or even death [2]. Rigid and semi-rigid optical stylets have gained utmost popularity in airway management in patients with limited neck extension, mouth opening, epiglottic cysts, thyroid tumors, and so on [3–7]. Thus, in the residency period and continued medical education, it is essential to induct training in optical stylets intubation technique awareness.

However, due to the pandemic of Coronavirus disease 2019 (COVID-19; severe acute respiratory syndrome coronavirus 2 [SARS-CoV-2]), traditional highly structured on-site skills training courses, such as hands-on workshops and simulation training, weren’t feasible to conduct offline [8]. It is imperative to develop new technical skills training models in medical education, which were less influenced by the pandemic of COVID-19.

With the improved development of interactive online education platforms and advanced electronic gears [9], it is possible to perform a real-time, remote demonstration of the required protocols. It is also convenient to record and share videos due to the popularity of video recording systems and the development of network technologies. The increased number of online training and webinars positively affected acquiring theoretical knowledge. In contrast, the feasibility and effectiveness of implementing online skills training (for example, training in tracheal intubation using optical stylets) remain to be explored.

It was proposed that a highly structured, proficiency-based training model helped improve clinical operating skills [10, 11]. A remote difficult airway management skills training project, named ‘All-in-one airway remote training program,’ was established in the Airway Management Training Center of Fudan University at the end of 2019 (www.linaatp.com). This study aims to evaluate the effect of launching a new remote hands-on training course regarding the intubation technique using an optical stylet.

## Methods

### Study design

This study was conducted as a before and after interventional study at Shanghai Eye & ENT Hospital of Fudan University. Ethical approval for this study (Ethical committee No. 2021008) was provided by the Ethical Committee of Shanghai Eye & ENT Hospital of Fudan University on January 15, 2021. All methods were performed following the 1975/1983 Helsinki declaration. Each participant provided written informed consent.

From February 2021 to October 2021, anesthesiologists were trained in a group of 10 to 12 participants. The competency of optical stylets intubation was evaluated by video recordings pre- and posttraining. Theoretical knowledge was examined by a pre-post multiple-choice questionnaire (MCQ) 3 days before and after training. Participants’ self-efficacy was assessed by using a self-reported pre-post questionnaire.

### Inclusion and exclusion criteria

Eligible residents and physicians met the following inclusion criteria: voluntarily participated in the training, had at least 1 year of practical experience in the department of anesthesiology, and had performed at least one-time tracheal intubation using optical stylets on the patient. Exclusion criteria included declining to submit the video recordings of intubation using optical stylets.

### Education and training of optical stylets intubation competency

**Fig. 1:**
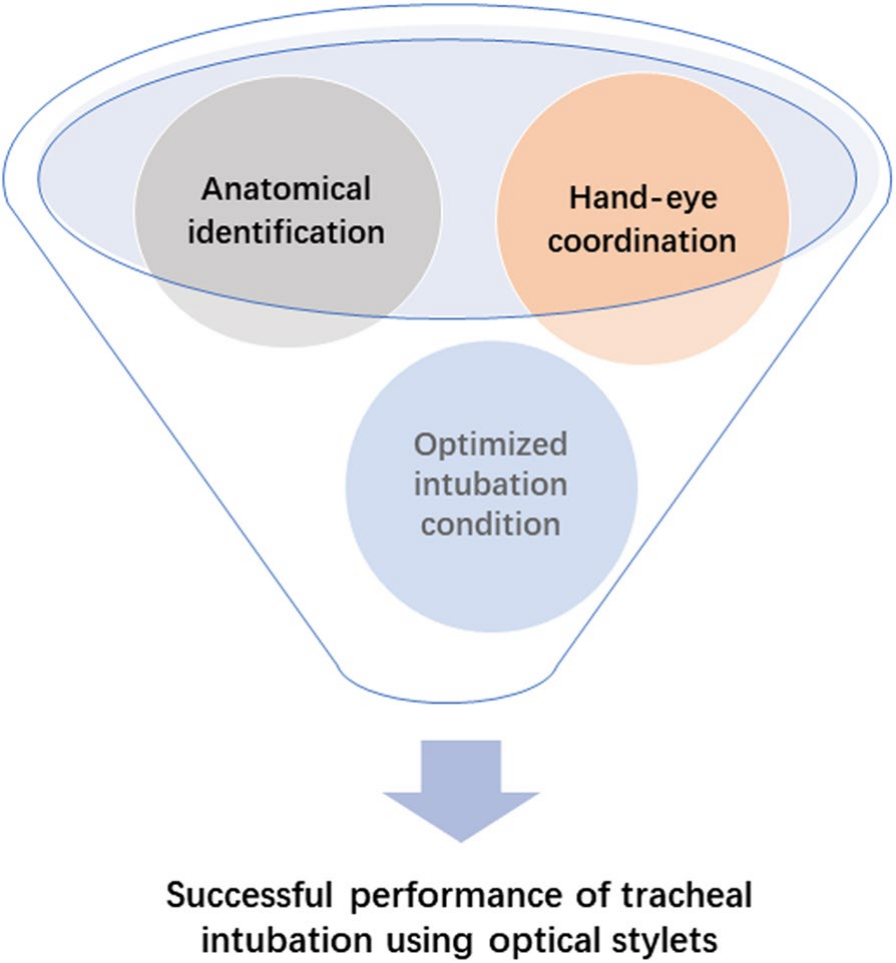
Critical skills required for tracheal intubation using optical stylets

**Fig. 2:**
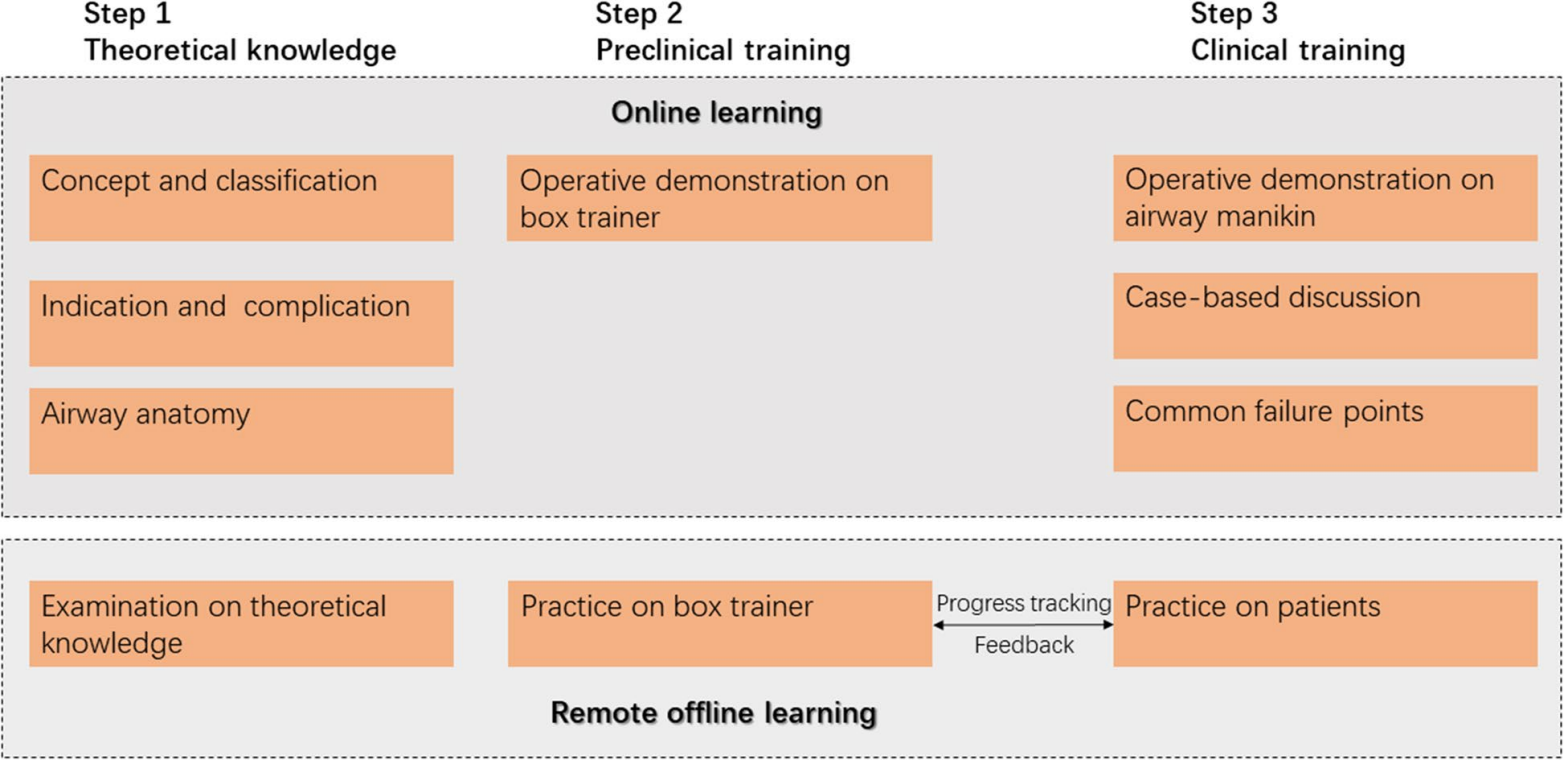
Flow diagram of three-step curriculum of optical stylets. The curriculum was implemented through online and remote offline learning

**Fig. 3:**
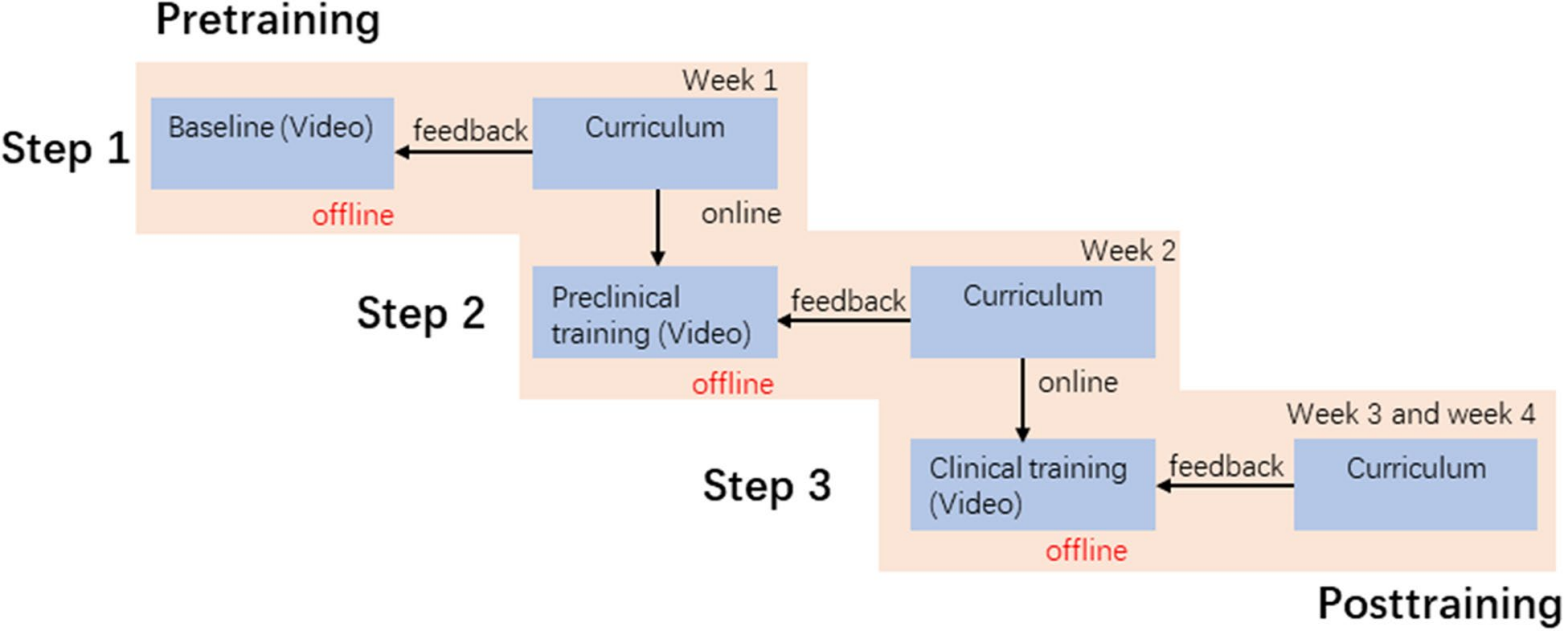
Contents of three-step optical stylets curriculum setup. The curriculum contents were splitted into theoretical knowledge, preclinical training, and clinical training. The curriculum was implemented through online or remote offline learning

Competency-based optical stylets training program focused on three critical skills: anatomical identification, hand-eye coordination, and optimized intubation condition (Fig. 1). The curriculum contents were splitted into three steps: theoretical knowledge, preclinical training, and clinical training through online and remote offline learning (Figs. 2 and 3). From Step 2, we gave feedback on trainees’ performance using the assessment scale as the essential educational tool during online coaching.

Step 1 consisted of fundamental theoretical knowledge, including concept and classification, indication and complication of optical stylets use, and airway anatomy. Theoretical knowledge and case-based discussion were communicated through a low-cost, commercial online live broadcasting application, ‘Tencent Meeting’ (Tencent, Shenzhen, China), in a live broadcasting room in the trainers’ center. By watching the interactive live broadcast, the trainees could communicate with the lecturers from any place without locational obligation, mimicking the face-to-face teaching environments.

**Fig. 4:**
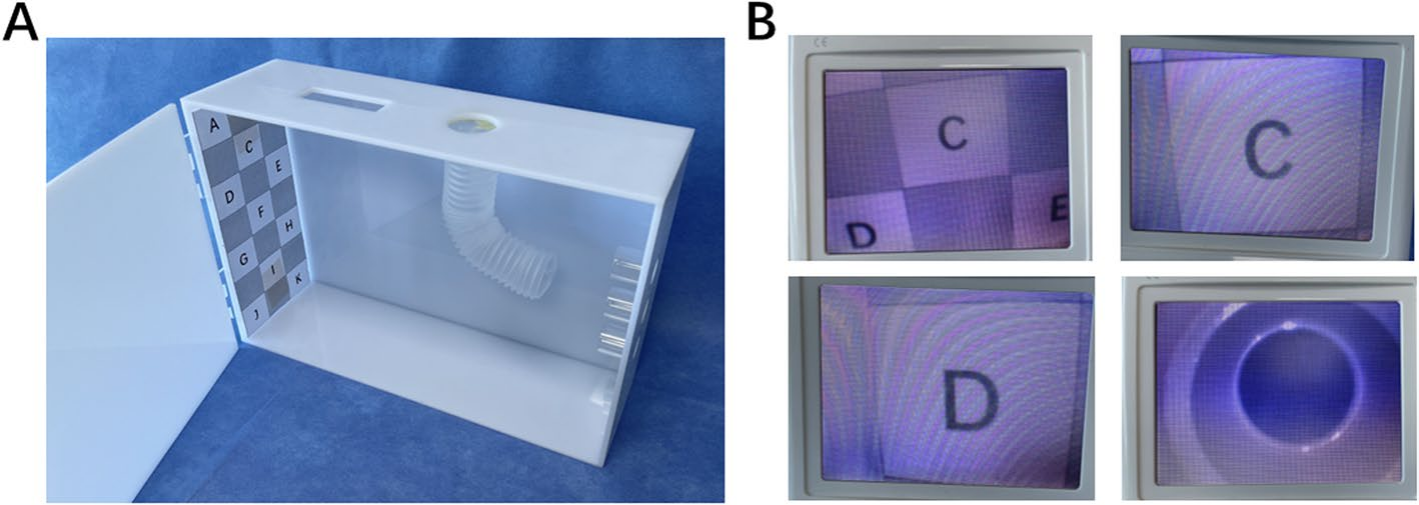
The schematic diagram of optical stylet training simulator. **A** Photograph of the optical stylet training simulator in our airway center. The model has been created using an acrylic material for better portability. **B** The images under the view of optical stylets

Step 2 consisted of hands-on preclinical training with a box trainer and video-based feedback. A novel portable training simulator created of acrylic material was designed for personal training (Fig. 4A) and shipped to every participant before training. The training simulator consists of a hand-made box with three hollow pipes on one side and English letters from A to K on the other side (Fig. 4B). It was designed to practice the essential operation of optical stylets, including forwarding and backward rotation direction and specific positioning. The operation demonstration was transmitted through the Tencent Meeting application. The trainees needed to practice using the optical stylets simulator for at least 1 hour and completed the designed procedures within 60 seconds before moving to the next training step.

Step 3 consisted of supervised clinical training and video-based feedback. Given that the novice practitioners needed about 20 cases to excel in the skill of optical stylets through the video-monitoring method, physicians were required to submit their 10th and 20th clinical cases. All procedures were performed on patients with normal airways and supervised by senior consultants to ensure patients’ safety. Each video consisted of a 20-minute one-on-one coaching session conducted by the supervisor, scheduled 0 to 5 days after intubation. All video-based feedback based on our assessment tool consisted of constructive guidance, identification of weaknesses and flaws, and positive reinforcement of the participants’ practice. The individual tutorials were realized through online live broadcasting software, a closed live chat function platform ‘WeChat’ (Tencent, Shenzhen, China), and cellular tools without requiring an in-person meeting.

### Competency assessment, knowledge testing, and self-efficacy evaluation

Competency assessment was carried out by video recordings of individual intubations using optical stylets on patients. The video recordings were taken in the participants’ hospitals. All patients with the Scale of American Society of Anesthesiologists grade > II and with difficult airway risks like the known history of difficult intubation, upper airway abnormalities, airway inflammation, tumors, abscesses, foreign bodies, known cervical spine injury; a small mouth (< 3 cm when open), a Mallampati score of ≥3, a body mass index of > 30 were excluded. An assessment tool (minimum score 0; maximum score 29) focused on anatomical identification, hand-eye coordination, and optimized intubation condition was designed to evaluate the skills based on the video recordings. The details are deeply briefed in the nominated table [see STable 1 in Additional file 1]. A higher intubation score indicates a better intubation performance. The validity and reliability of this assessment tool were resolute in our study, shown in Additional file 1. All videos were randomly numbered. Two raters (including the experienced consultants) blinded to the participant’s identity scored the videos and recorded the intubation time. Then the results were calculated on the averages and interrater reliability assessment.

Academical knowledge testing was performed using a MCQ exam of 20 questions focusing on indications, intubation techniques, and complications of optical stylets use. The MCQ exam was designed based on currently available best practice literature and validated by four senior anesthesiologists by a modified Delphi technique. Senior anesthesiologists rated each item, which was retained if the consensus was reached with at least 80% of the clinical experts’ voting [12]. At the end of the process, the final tool consisted of 20 items with a maximum acquiring score of 100 points. Then the questions were trialed with four anesthesiologists to ensure that the MCQ exam was understandable and consistent with the training content.

A questionnaire performed self-efficacy evaluation, an individual’s confidence in the ability to complete a specific task [13]. Self-efficacy was also helpful in mediating the reduction of the knowledge-behavior gap among health care workers [14]. Questions were asked about participants’ ability to use optical stylets on a 5-item scale which ranged from 1 (strongly disagree) to 5 (strongly agree). Responses of “not applicable” for any item were excluded. The self-efficacy was also designed based on the previous study [13], and the final tool consisted of 7 items.

### Outcomes

The primary study outcome was competency measured by the assessment tool according to pre-post video recordings. The time to successful intubation was recorded (from insertion to removal of the stylet). The intubation quality score (score per minute) was also calculated based on a previous study [15]. The secondary outcomes were the scores of the pre-post MCQ exam and self-efficacy evaluated by the pre-post questionnaire.

### Sample size calculation

In the educational field, an adequate sample size of above 1.0 SD is considered relevant and significant for an educational intervention reported in the previous study [16]. Thus, assuming such an effect size of 1.0 SD, a sample of 15 participants was sufficient to achieve a discriminating power of 80% with a 2-sided alpha level of 5%. Considering airway management skill is essential for anesthesiologists’ continuing education, we decided to include all eligible participants during the recruitment period. This also would allow us to ensure a sufficient sample size despite the likelihood of participants dropping out.

### Statistical analysis

Statistical analyses were performed using SPSS 19.0 (Chicago, IL, USA). The normal distribution of the outcome variables was analyzed using the Shapiro–Wilk test. Interrater reliability was assessed using Pearson product-moment correlation coefficient. The scores of intubation performance and self-efficacy were expressed as mean ± SD using a paired t-test. The MCQs questionnaire was presented as median [IQR] and was compared using the Wilcoxon signed-rank test. The characteristics of participants were expressed as numbers and percentages. *P* < 0.05 was considered statistically significant.

This manuscript was prepared according to the Strengthening the Reporting of Observational Studies in Epidemiology (STROBE) statement [17].

## Results

### Baseline participant characteristics

**Fig. 5:**
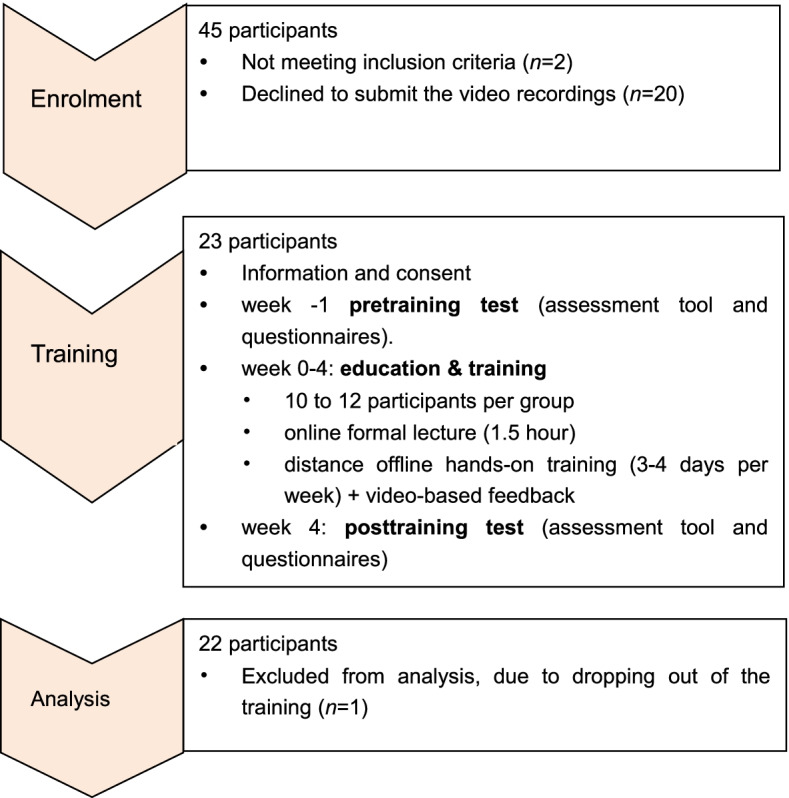
Flow chart

From February to October 2021, among 45 anesthesiologists who were invited to the study, 22 remained as demonstrated in Fig. 5 (8 females, 14 men, mean age of 33.5 years). Overview of participants regarding gender, age, professional level, grade of the hospital, working years, and the type of optical stylets are shown in Table 1.

**Table 1:** Baseline of participants’ characteristics in the training

Measure	Items	Frequency	Percent (%)
Gender	Male	8	36
	Female	14	64
Age (year)	20-30	5	23
	30-40	15	68
	40-50	2	9
	Over 50	0	0
Grade of hospital	Grade II	6	27
	Grade III	16	73
Experience in anesthesiology (years)	1-3 years	3	14
	3-5 years	7	32
	5-10 years	7	32
	Over 10 years	5	22
Performed intubation using optical stylets (times)	1-5	9	41
	6-10	3	14
	11-50	6	27
	Over 50	4	18
Classification of optical stylets	rigid	8	36
	semi-rigid	14	64

Values are expressed as numbers and percentages

### The outcome of training and scores of intubation skills

**Fig. 6:**
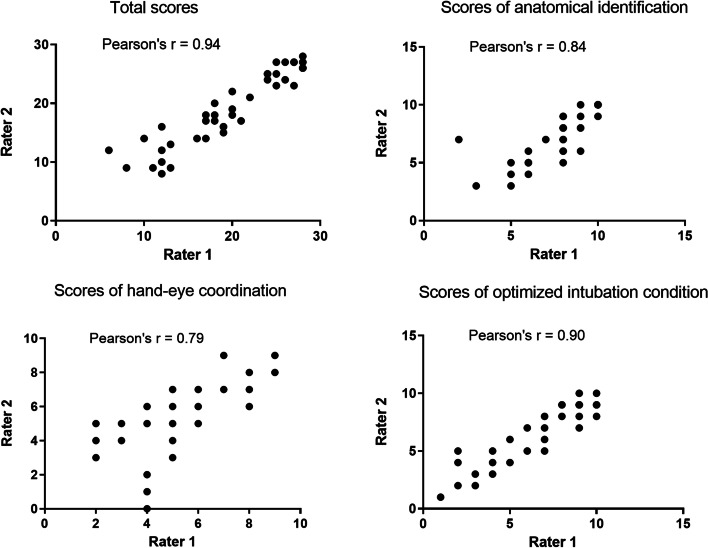
The interrater reliability of the assessment tool for intubation using optical stylets. The interrater reliability concerning the total scores based on 44 video recordings was good, Pearson’s *r* = 0.94. Reliability was highest for optimized intubation condition, Pearson’s *r* = 0.90, and the anatomical identification, Pearson’s *r* = 0.84, somewhat lower concerning hand-eye coordination, Pearson’s *r* = 0.79

A total of 44 videos were reviewed and scored. The intubation performance was assessed by a designed assessment tool (STable 1). The Pearson product-moment correlation coefficient of the interrater reliability is 0.94 (Fig. 6). The construct and content validity of the assessment tool has been conducted, and the results were shown in STable 2 and STable 3.

The average score of overall intubation performance was significantly improved posttraining compared to the pretraining period (25.3 ± 2.6 vs. 14.6 ± 3.7, *P* < 0.0001). The average score for anatomical identification, hand-eye coordination, and the optimized intubation condition increased significantly (*P* < 0.0001, Table 2).

**Table 2:** Participants’ pre and posttraining competency scores of tracheal intubation using optical stylets

	Pretraining	Posttraining	***P*** value
	(***n*** = 22)	(***n*** = 22)	
**Total score (29 is the full mark)**	14.6 ± 3.7	25.3 ± 2.6	< 0.0001
Anatomical identification	6.0 ± 1.4	9.5 ± 0.8	< 0.0001
Hand-eye coordination	4.3 ± 1.4	7.8 ± 0.9	< 0.0001
Optimized intubation condition	4.3 ± 2.0	8.1 ± 1.7	< 0.0001
**Intubation time, second**	54.7 ± 39.1	44.5 ± 19.7	0.130
**Quality score (ratio of score to time)**	0.44 ± 0.32	0.65 ± 0.25	0.005

The values are mean ± SD. Intubation time, second: from insertion of an optical stylet into the oral cavity and ended when the stylet was drawn from the oral cavity

Interestingly, the scores in hand-eye coordination and optimized intubation condition domain improved to a similar degree between the less (performed 1-50 optical stylets) and more experienced trainees (performed > 50 optical stylets). However, the degree of improvement of anatomical identification scores was lower in the more experienced trainees than that in the less ones (*P* = 0.016, Table 3).

**Table 3:** The changes in participants’ pre and posttraining competency scores of tracheal intubation using optical stylets

	Performed 1-50 optical stylets (***n*** = 18)	Performed over 50 optical stylets (***n*** = 4)	***P*** value
**Total score (29 is the full marks)**	11.3 ± 4.2	7.9 ± 1.9	> 0.05
Anatomical identification	3.9 ± 1.6	1.6 ± 0.6	0.016
Hand-eye coordination	3.6 ± 1.5	2.9 ± 1.0	> 0.05
Optimized intubation condition	3.8 ± 2.5	3.4 ± 0.4	> 0.05
**Intubation time, second**	−13.7 ± 31.7	5.9 ± 13.2	> 0.05
**Quality score (ratio of score to time)**	0.2 ± 0.3	0.1 ± 0.3	> 0.05

The values are mean ± SD. Intubation time, second: from inserting the stylets into and out of the mouth

The correct answers for MCQs increased from 58.2 ± 8.2% before training to 85.2 ± 7.2% shortly after training (*P* < 0.0001). Forty-four questionnaires were returned and analyzed. There were significant improvements in self-efficacy scores for all seven subitems compared with pretraining (all *P* < 0.0001, except one *P* = 0.0003, Table 4), with the sum of all items increasing from 2.5 ± 1.2 pretraining to 4.4 ± 0.6 posttraining (*P* < 0.0001, Table 4).

**Table 4:** Participants’ pre and posttraining self-efficacy scores of tracheal intubation using optical stylets

No	Self-efficacy item: ‘I am confident in my ability to …’		Pretraining	Posttraining	***P*** value
			*n* = 22	*n* = 22	
1.	Know about oropharyngeal anatomy under the view of optical stylets		2.6 ± 0.9	4.6 ± 0.5	< 0.0001
2.	Have an awareness of identification of anatomy during intubation using optical stylets		2.5 ± 1.3	4.7 ± 0.5	< 0.0001
3.	Understand the concept of operating gently and avoiding impacts on fragile surroundings		2.7 ± 1.4	4.7 ± 0.5	< 0.0001
4.	Have a high success rate of intubation using optical stylets		2.2 ± 1.1	4.1 ± 0.6	< 0.0001
5.	Grasp the operational essentials of intubation using optical stylets		2.7 ± 1.7	4.2 ± 0.5	0.0003
6.	Have the confidence of intubation using optical stylets		2.2 ± 0.9	4.4 ± 0.5	< 0.0001
7.	Solve the intubation problems related to video laryngoscope through optical stylets		2.6 ± 0.7	4.2 ± 0.6	< 0.0001
Mean ± SD			2.5 ± 1.2	4.4 ± 0.6	< 0.0001
Scoring	1	2	3	4	5
	Strongly disagree	Disagree	Neutral	Agree	Strongly agree

The values are mean ± SD. Not applicable = not included. Degree of agreement of the questionnaires on a 5-point Likert scale. Likert scale ranging from 1 (strongly disagree) to 5 points (strongly agree) per item

## Discussion

Our study has shown that the competency of optical stylets on patients with normal airways significantly increased after remote training. Among these, three critical skills were improved considerably, including anatomical identification, hand-eye coordination, and optimized intubation conditions. In addition, participants’ knowledge and self-efficacy in using optical stylets were also significantly improved. Overall, this remote training model is an effective teaching technique for optical stylets intubation training.

Optical stylets not only offer effective airway management aid [3–7], but also can reduce intubation complications such as hoarseness and sore throat compared with laryngoscope [18]. Skillful usage of optical stylets is essential for the anesthesiologist. However, the lack of experienced faculty, inconsistent availability of equipment, and intubation methods differing from video laryngoscopy can be obstacles. To our knowledge, this is a novel study investigating the efficiency of a remote airway management project to improve optical stylets intubation competency.

It is challenging to implement airway management technique training during the COVID-19 pandemic. In this study, we guaranteed the training effectiveness through several strategies. One one hand, we have adopted a good curriculum structure from simple to complex ideas in remote training, from basic theoretical to preclinical and clinical training. On the other hand, participants acquired hands-on skill proficiency through deliberate practice with video-based feedback instead of face-to-face guidance. Furthermore, we ensured patients’ safety by enrolling experienced participants, supervised operations on patients with normal airways, and provided instant feedback using ‘WeChat’. Considering that not all trainees had access to airway manikins, and all participants had at least 1 year of anesthesiology experience with basic upper airway anatomy knowledge, airway manikins’ performance was not mandatory. This flexible skill training model could be extended to other airway management skills training under the pandemic.

We designed the training curricula based on the learning curve of optical stylets intubation and our center’s training experience. The expertise criterion was defined as the intubation experience of > 100 optical stylets, according to a previous study regarding rigid bronchoscopies [19]. The three critical techniques as an expert are recognizing the anatomical landmark, to be hand-eye coordination and optimizing intubation conditions. Instead of speeding up the intubation time, we encouraged the participants to slow the intubating speed and put their awareness on avoiding contact with surrounding mucosal tissue. With the video monitor during the intubating procedure, the “avoiding contact” technique highly reduces the incidence of injury, bleeding, and mass rupture caused by blind approach [20].

In recent years, many studies have presented and assessed the effectiveness of remote medical education. Among which, only several studies reported remote learning regarding hands-on clinical skills, focusing on training effect in preclinical settings instead of real clinical ones [21–23]. However, the skills’ evaluation, including navigational skills, decision-making, team cooperation, and anatomical identification, could hardly be assessed based on the remote simulator [23]. In this study, our training program focused on improving the optical stylets intubation skill and tested the effectiveness in clinical settings.

In this remote training circumstance, we used video recordings for performance evaluation and improvement instead of direct observations in the operation room. Beneficially, video recording is an emerging approach to evaluate health workers’ performance, such as neonatal resuscitation [24–26] and surgery skills [27, 28], thus ultimately enlightening patient safety. The video-recorded clinical performance is cost-effective and noninvasive to the performed procedure. The researchers would be able to secure highly observational and essential data of real-time happenings [29], and directs it towards individualized goal-directed learning [30]. The results of our study were consistent with previous studies [24–26].

The assessment tool was designed specifically for this study with assurance of its validity and reliability because no previously validated tools were available. Our assessment tool can aid in recognizing the most experienced subject compared to novices, making it a progressive tool.

Our study has some limitations. First, the number of participants is limited. We minimized the number of tutor participants in the ‘classroom’ and allowed more interaction for the trainees with peers and instructors. Introducing a larger scale, remote training needs to be carefully planned and organized [31]. Second, technical skills may decline noticeably after three to six months [32, 33], and skill retention should be tested in the sustainability phase. Third, our study was a before and after research, not a parallel randomized controlled trial of two groups. A randomized controlled trial should be designed to prove the effectiveness of remote hands-on airway management skills training.

## Conclusion

In conclusion, this study confirms the value of an advanced hands-on technical skill remote training model for anesthesiologists to learn optical stylets intubation technique. Furthermore, this training model may be feasible for other airway management skills training under the COVID-19 pandemic.

## Supplementary Information

**Additional file 1.**

## Data Availability

All data and additional file are available upon request from the corresponding author.

## Abbreviations

COVID-19: Coronavirus disease 2019
MCQ: Multiple-choice questionnaire
