# Supplementary material for: Improved skill for tracheal intubation using optical stylets through remote training model: a before and after interventional study
Source: BMC Med Educ. 2022 Sep 10;22:668. doi: 10.1186/s12909-022-03715-x (PMC9462891; doi:10.1186/s12909-022-03715-x)
Supplement: Supplementary file 1 — Additional file 1. [file 12909_2022_3715_MOESM1_ESM.docx]

**Supplementary material 1：The content and construct validity of tracheal intubation using optical stylet skill-specific assessment tool**

1. **Method**

**1.1 Content validity**

A team of experts in the Airway Management Training Center of Fudan University performed the first drafting of the assessment tool of the individual items according to a previous study assessing clinical bronchoscopy performance [1]. A modified Delphi methodology was used to select the best steps to establish content validity by 10 expert clinical anesthesiologists from different hospitals. Experts rated each item on a 9-point Likert-type scale (1 = not important and 9 extremely important). The element was retained if the consensus was reached, with at least 80% of the clinical experts voting at 7 or above [2]. At the end of the process, the final tool was classified into three categories and consisted of 17 items with a maximum score of 29 points as follows **(STable 1):**

**STable 1** Tracheal intubation using optical stylet skill-specific assessment tool

|  | **Items** | **Criteria for mark allocation** | | |
| --- | --- | --- | --- | --- |
|  |  | **0** | **1** | **2** |
| **Anatomical identification** | Uvula | False | Uncertain but correct | Correct |
|  | Epiglottis | False | Uncertain but correct | Correct |
|  | [The apex of the arytenoid cartilage](http://dict.youdao.com/w/eng/apex_of_arytenoid_cartilage/#keyfrom=dict.phrase.wordgroup), false vocal cord | False | Uncertain but correct | Correct |
|  | Anterior commissure, posterior commissure | False | Uncertain but correct | Correct |
|  | Annulus trachea | False | Uncertain but correct | Correct |
| **Hand-eye coordination** | Scope centering | Frequent pointing away from the center | Scope-centered most of the time | Scope always centered |
|  | Correctly stabilizes the optical stylet | Not at all | Optical stylet stabilized most of the time | Optical stylet always stabilized |
|  | The correct distance between the tip of stylet and tube (0.3-0.5 cm) | No | Yes | / |
|  | Rotates the tube centered so that the tip faces [glottis](http://dict.youdao.com/w/glottis/#keyfrom=E2Ctranslation) | No | Yes | / |
|  | Correctly places the tube into [glottis](http://dict.youdao.com/w/glottis/#keyfrom=E2Ctranslation) and removes the stylet | No | Yes | / |
|  | Inserts the tube [smoothly](http://dict.youdao.com/w/smoothly/#keyfrom=E2Ctranslation) | No | Yes | / |
|  | Successful intubation for the first time | No | Yes | / |
| **Optimized intubation condition** | Creating space for [oral cavity](http://dict.youdao.com/w/oral%20cavity/#keyfrom=E2Ctranslation) | Poor | Accepted | Good |
|  | Clarity of fields under the screen | Poor | Accepted | Good |
|  | Secretions | Frequent | Few | None |
|  | Collisions on [mucosa](http://dict.youdao.com/w/mucosa/#keyfrom=E2Ctranslation) or [glottis](http://dict.youdao.com/w/glottis/#keyfrom=E2Ctranslation) | Frequent | Few | None |
|  | Blood | Frequent | Few | None |

The assessment tool focuses on three critical skills: anatomical identification, hand-eye coordination, and optimized intubation conditions (1-29 points, 29 is full mark). A higher intubation score indicates better intubation performance.

**1.2 Construct validity**

30 anesthesiologist volunteers from different hospitals were selected in three categories: 10 novices (performed 0-5 optical stylet, but at least 100 video laryngoscopic intubations), 10 intubators with intermediate experience (performed 11-50 optical stylet intubations), and 10 experts (performed >100 optical stylet intubations). Each subject performed optical stylet intubation on a patient in their hospitals. Two independent raters scored videotaped intubation procedures. One rater scored the intubations twice. The tool was assessed for construct validity.

**2. Results**

**2.1 Demographic data of the participants**

The study sample included 30 volunteers in anesthesiology apartment from different hospitals. A total of 30 participants were recruited at novice, intermediate, and expert levels based on performed optical stylets intubation number with 10 person each group. **STable 2** shows the participants' demographic data (age, sex, grade of hospital, clinical role, working experience in anesthesiology).

**STable 2 Demographics of the participants n (%)**

| Category | Novice  (n=10) | Intermediate (n=10) | Expert  (n=10) | *P* value |
| --- | --- | --- | --- | --- |
| Sex |  |  |  |  |
| Male | 4 (40) | 3(30) | 6 (60) | >0.05 |
| female | 6 (60) | 7 (70) | 4 (40) |  |
| Age (year) |  |  |  |  |
| 21–30 | 2 (20) | 2 (20) | 1 (10) | >0.05 |
| 31–40 | 7 (70) | 7 (70) | 6 (60) |  |
| 41-50 | 1 (10) | 1 (10) | 3 (30) |  |
| Over 50 | 0 (0) | 0 (0) | 0 (0) |  |
| Grade of hospital |  |  |  |  |
| Grade II | 3 (30) | 2 (20) | 1 (10) | >0.05 |
| Grade III | 7 (70) | 8 (80) | 9 (90) |  |
| Clinical role |  |  |  |  |
| Resident | 2 (20) | 2 (20) | 1 (10) | >0.05 |
| Attending | 6 (60) | 8 (80) | 7 (70) |  |
| Professor | 2 (20) | 0 (0) | 2 (20) |  |
| Working experience in anesthesiology apartment |  |  |  |  |
| 1-3 years | 0 (0) | 2 (20) | 1 (10) | >0.05 |
| 3–5 years | 4 (40) | 3 (30) | 2 (20) |  |
| 5-10 years | 4 (40) | 3 (30) | 2 (20) |  |
| Over 10 years | 2 (20) | 2 (20) | 5 (50) |  |

**2.2 Construct validity**

The mean total scores were significantly different among the three groups, and post hoc tests revealed that the intermediate group performed significantly better than the novice operator (*P* < 0.001, **STable 3**), and the expert operators also performed significantly better than the intermediate operators (*P* < 0.001, **STable 3**). The results of all subitems including anatomical identification, hand-eye coordination, and optimized intubation condition, were also shown in **STable 3.** Furthermore, the data demonstrated that intermediate and expert operators spent less time completing intubation than novice operators (*P* = 0.005 and *P* = 0.002, respectively); however, no difference was found between intermediate and expert **(STable 3)**.

**STable 3 Comparisons among the individual groups concerning total score, anatomical identification, hand-eye coordination, optimized intubation condition and intubation time (n=10)**

| **Group** | **Score** | | | | **Intubation time, second** |
| --- | --- | --- | --- | --- | --- |
|  | **Total score**  **(29 points)** | **Anatomical identification (10 points)** | **Hand-eye coordination (9 points)** | **Optimized intubation condition (10 points)** |  |
| **Novice** | 11.4±2.4 | 5.2±1.3 | 3.5±1.2 | 2.9±1.1 | 92.2±48.6 |
| **Intermediate** | 16.2±2.9 | 6.1±1.4 | 5.0±1.2 | 5.2±1.9 | 42.2±26 |
| Novice vs Intermediate  P value | ＜0.001 | 0.269 | 0.007 | 0.004 | 0.005 |
| **Expert** | 26.0±1.2 | 9.5±0.8 | 8.2±0.6 | 8.4±1.2 | 35.6±11.9 |
| Novice vs Expert  P value | ＜0.001 | ＜0.001 | ＜0.001 | ＜0.001 | 0.002 |
| Intermediate vs Expert  P value | ＜0.001 | ＜0.001 | ＜0.001 | ＜0.001 | 0.893 |

The scores are shown as mean ± SD. The extent of intubation was recorded as the 3 total number of attempts or 3.5 minutes in duration (An attempt was defined as from insertion of an optical stylet into the oral cavity and ended when the stylet was drawn from the oral cavity).

**Reference**

1. Konge L, Larsen KR, Clementsen P, Arendrup H, von Buchwald C, Ringsted C: Reliable and valid assessment of clinical bronchoscopy performance. Respiration. 2012; 83(1):53-60.

2. Gogovor A, Zomahoun HTV, Ben Charif A, McLean RKD, Moher D, Milat A, Wolfenden L, Prévost K, Aubin E, Rochon P *et al*: Essential items for reporting of scaling studies of health interventions (SUCCEED): protocol for a systematic review and Delphi process. Syst Rev. 2020; 9(1):11.
